# Microsatellite Support for Active Inbreeding in a Cichlid Fish

**DOI:** 10.1371/journal.pone.0024689

**Published:** 2011-09-30

**Authors:** Kathrin Langen, Julia Schwarzer, Harald Kullmann, Theo C. M. Bakker, Timo Thünken

**Affiliations:** 1 Institute for Evolutionary Biology and Ecology, University of Bonn, Bonn, Germany; 2 Zoologisches Forschungsmuseum Alexander Koenig, Bonn, Germany; 3 Zentrum für Didaktik der Biologie, University of Münster, Münster, Germany; Lund University, Sweden

## Abstract

In wild animal populations, the degree of inbreeding differs between species and within species between populations. Because mating with kin often results in inbreeding depression, observed inbreeding is usually regarded to be caused by limited outbreeding opportunities due to demographic factors like small population size or population substructuring. However, theory predicts inclusive benefits from mating with kin, and thus part of the observed variation in inbreeding might be due to active inbreeding preferences. Although some recent studies indeed report kin mating preferences, the evidence is still highly ambiguous. Here, we investigate inbreeding in a natural population of the West African cichlid fish *Pelvicachromis taeniatus* which showed clear kin mating preferences in standardized laboratory experiments but no inbreeding depression. The presented microsatellite analysis reveals that the natural population has, in comparison to two reference populations, a reduced allelic diversity (A = 3) resulting in a low heterozygosity (H_o_ = 0.167) pointing to a highly inbred population. Furthermore, we found a significant heterozygote deficit not only at population (F_is_ = 0.116) but also at subpopulation level (F_is_ = 0.081) suggesting that inbreeding is not only a by-product of population substructuring but possibly a consequence of behavioral kin preferences.

## Introduction

In wild animal populations, the levels of inbreeding are highly variable [1]. Close inbreeding in natural populations has been reported, for example, in the eusocial naked-mole rat [2]–[4], in social spiders [5] and in insects [6], [7]. Inbreeding can be caused by demographic factors like small population size, limited dispersal or population substructuring, and by preferential mating among relatives [8]. Although inbreeding can increase the inclusive fitness of an individual [9]–[17], maintain co-adapted gene complexes or local adaptations [18], the costs of inbreeding usually seem to override these benefits and therefore mating patterns are normally characterized by inbreeding avoidance [19]–[23]. When inbreeding occurs over multiple generations, the frequency of homozygotes is increased [24], [25], often resulting in a decline of fitness in inbred offspring [26]. Furthermore, inbred populations may suffer in the long term from reduced allelic diversity for instance because of reduced potential to response to ecological changes. Although inbreeding does not affect allele frequencies directly, in combination with selection and drift it may do so. Inbreeding depression may reduce population size. Small, isolated populations in particular lose genetic diversity faster than large populations due to genetic drift. Inbreeding depression has been shown in several studies of wild populations (reviewed by [1], [27], [28]). Thus, observed inbreeding in natural populations is thought to be mainly caused by limited outbreeding opportunities.

However, the costs of inbreeding, i.e. the strength of inbreeding depression can vary across taxa, populations and environments [1], [27], [29], [30]–[32]. Inbreeding depression is expected to become less severe when inbreeding is persistent [33], [34]. The reason for this is purging. It is defined as selection against deleterious, recessive alleles in a population affected by inbreeding [35], so that inbreeding causes only little or no reduction in fitness [1]. Several studies actually showed that deleterious inbreeding effects might be reduced due to purging [29], [32], [36]–[38]. In this case, short term benefits of inbreeding might exceed its costs and inbreeding tolerance [39] or even mating preferences for kin might evolve [12].

*Pelvicachromis taeniatus*, a socially monogamous cichlid fish with dedicated biparental brood care [40], is one of the rare examples of inbreeding preferences. In standardized laboratory experiments, when given the choice simultaneously between kin and non-kin of the opposite sex, both males and females preferred kin over non-kin as mating partners [12], [41]. This active inbreeding behavior appears to be adaptive because related parents provide better care and inbreeding individuals might increase their inclusive fitness [12]. Furthermore, inbreeding does not seem to be associated with high costs: at least under laboratory conditions, in- and outbred offspring did not significantly differ in terms of survival or growth rate [12], traits known to be highly affected by inbreeding in fishes [42]. The observed lack of inbreeding depression might be the result of purging of deleterious alleles from natural populations, provided that inbreeding also occurs regularly in nature.

The aim of the present study was to estimate the level of inbreeding in the original wild population, i.e. *P. taeniatus* from the Moliwe river in Cameroon. According to the laboratory findings, we expected to find a highly inbred wild population. The level of inbreeding was estimated indirectly by examining microsatellite heterozygosity. First, we compared the Moliwe population with another *P. taeniatus* population and a population of the closely related species *Pelvicachromis pulcher* in order to estimate the reliability of the chosen microsatellite loci and to examine population-wide differences in genetic diversity. Second, we analyzed the Moliwe population in detail. As population substructuring can affect kin-biased mating patterns, we first checked for subpopulations and subsequently examined heterozygote deficits within subpopulations as indicator for kin mating.

## Materials and Methods

### Ethics statement

For this study only a small part of the caudal fin was clipped. Fins fully regenerate within a few weeks. According to §5 of the german animal welfare act (BGBI. I 1206, 1313) no approval is necessary and an anaesthetization is not necessary, when at a comparable procedure in humans an anaesthetization usually is not done or when the pain related with the procedure is less than the negative effects of the anaesthetization. The study conforms to the animal behaviour society guidelines for the use of animals in research as well as to the legal requirements of Germany.

### Study species, study site and sampling

**Figure 1:**
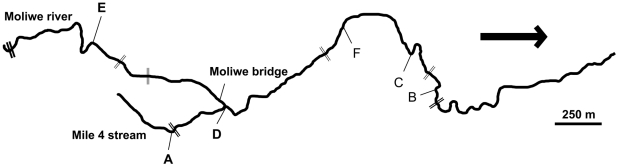
Map of the Moliwe river system. The six sampling sites are marked A–F. The Moliwe river system consists of the Moliwe river and the smaller Mile 4 stream. Double bars indicate physical barriers like waterfalls and rapids (the thickness of the symbol corresponds to the extent of the migration barrier). The arrow indicates the direction of the water flow. The map is based on GPS data (recorded with Magellan explorist 100 GPS Receiver).

*Pelvicachromis taeniatus* occurs in drainages from Nigeria to Cameroon. The species shows a conspicuous size and color dimorphism [43], [44]. Males (6–8 cm) are larger than females (4–5.5 cm). The study population (Moliwe) originates from the Moliwe river and a smaller stream (Mile 4 stream), which flows into the Moliwe river (Figure 1). The river system is located in southwestern Cameroon (04 03.669 N/009 14.736 E) and crosses the street from Limbe to Kumba between the villages Moliwe and Mile 4. It is geographically separated from surrounding river systems through the foothills of the Mount Cameroon massif and empties into a river that inhabits no other *Pelvicachromis*, isolating the *P. taeniatus* population from Moliwe. Several waterfalls of different height and rapids subdivide the river into different stretches.

In June 2007, a total of 200 specimens were collected at five sampling sites (B–F) distributed along the Moliwe river (Figure 1) and at one sampling site in the Mile 4 stream (A) using hand nets. At each sampling site individuals were sampled within an area of 20 to 30 m. A small part of the caudal fin was clipped and all fish were released afterwards. Forty tissue samples were taken at sites A–D and 34 at F. At sampling site E only six individuals were caught. For comparison, nine wild-caught specimens of *P. taeniatus* and 31 wild-caught specimens of *P. pulcher* from two populations in Nigeria were obtained from a commercial aquarium-fish importer (Mimbon-Aquarium, Cologne, Germany) in April 2008. The fish were maintained at the Institute for Evolutionary Biology and Ecology in Bonn, Germany where fin clips of each individual were taken. All tissues were stored in 2 ml tubes containing 99.8% ethanol at −20°C.

### DNA extraction and cross species amplification of microsatellite primers

DNA extraction was conducted using the QIAGEN DNeasy Blood and Tissue Kit (QIAGEN). The DNA concentration of each sample was measured using the spectrophotometer NanoDrop™ 1000 (Thermo SCIENTIFIC). DNA was diluted with distilled water to a uniform concentration of 25 ng/µl.

Because no microsatellites had been yet developed for *P. taeniatus*, it was necessary to first assay a large number of potential primers. Based on a linkage map of 525 microsatellite loci available from *Oreochromis niloticus* [45], a subset of 169 microsatellite loci were selected. These microsatellites are all dinucleotide repeats of the type (CA)_n_ and have been successfully cross-species amplified in other cichlid studies [46], [47]. Assuming that the location of the loci on the chromosomes is similar in *Pelvicachromis* and *O. niloticus*, loci were chosen from different chromosomes in order to assure representative coverage of the whole genome. With respect to the location on a single chromosome, loci were chosen haphazardly. Furthermore, an appropriate distance between loci on the same chromosomes was considered, to ensure independency of markers. Primer pairs of the subset were tested on two samples of *P. taeniatus* Moliwe (one male and one female). For PCR the QIAGEN Multiplex PCR kit (QIAGEN) was used, comprised of a Master Mix including HotStarTaq® DNA polymerase, Multiplex PCR Buffer and dNTP Mix. PCR amplifications for first primer tests were performed using 10 µl reaction volumes containing 5 µl multiplex mix, 0.8 µl forward (50 pmol/µl) and 0.8 µl reverse (50 pmol/µl) primer, 1 µl DNA (25 ng/µl) and 2.9 µl HPLC water. PCR amplifications were carried out in a GeneAmp 2720 Thermo Cycler (Applied Biosystems). When PCR products showed a well-defined, strong white single band on a 1.5% agarose gel (this was the case for 44 microsatellite loci), primers were chosen for further analyses.

### Genotyping

The tailed primer method [48] provides an economic opportunity for fluorescent labeling of PCR fragments and was applied on the 44 microsatellites. Thirty-four out of the 44 tested microsatellites revealed analyzable products on the capillary sequencer (CEQ 8800 Genetic Analysis System, Beckman Coulter) and were checked for polymorphic bands. Initially, 36 individuals of *P. taeniatus* of the Moliwe population (six of each sampling site), six individuals of *P. taeniatus* from Nigeria and five individuals of *P. pulcher* from Nigeria were tested for polymorphism. A blank sample was included to check for contamination. Seventeen loci were polymorphic within at least one of the populations and were chosen for population genetic analyses of the total sample (Table S1).

The following universal fluorescent dyes and tail primers were used: M13-tail with dye label D2 (5′-[D2-PA]TGTAAAACGACGGCCAGT-3′), T7-tail with dye label D3 (5′-[D3-PA]TAATACGACTCACTATAG-3′) and Sp6-tail with dye label D4 (5′-[D4-PA]GATTTAGGTGACACTAT-3′). Forward primers were ordered with a tail corresponding to the specific fluorescent labeled primers (M13 tail: 5′-TGTAAAACGACGGCCAGT-3′, T7 tail 5′-TAATACGACTCACTATAG-3′, Sp6 tail 5′- GATTTAGGTGACACTAT-3′).

PCR reactions were multiplexed with up to three microsatellite loci in one PCR. Amplifications were carried out in a total volume of 10 µl containing 5 µl multiplex mix (Qiagen Multiplex PCR kit), 1 µl DNA (25 ng/µl), 0.1–0.2 µl forward primers (2.5 pmol/µl), 0.2–0.4 µl reverse primers (5 or 10 pmol/µl) and 0.2–0.4 µl labeled primers (1–10 pmol/µl depending on mix, locus and dye) and HPLC water (to bring to volume). Primer concentrations depended on the strength of locus amplification and dye signal in the multiplexed PCR [49]. Loci were amplified using the following PCR profile: preheating at 95°C for 15 min, 30 cycles of 60 s at 94°C, 45 s at 58°C, 60 s at 72°C, 8 cycles of 60 s at 94°C, 45 s at 53°C, 60 s at 72°C and a final extension cycle of 30 min at 72°C.

Genotypes were scored on a CEQ 8800 Genetic Analysis System (Beckman Coulter). PCR products were diluted with 200 µl distilled water. One µl of template was mixed with 0.1 µl of GenomeLab™ DNA Size Standard 400 (Beckman Coulter) and 29 µl of GenomeLab™ Sample Loading Solution (SLS, contains formamide) (Beckman Coulter). The CEQ 8800 software version 9.0.25 (Beckman Coulter) was used to analyze the allele sizes of the microsatellites. To generate the data format for commonly used population genetics software the Excel Microsatellite Toolkit [50] was applied.

### Minimizing and identifying allele scoring errors in microsatellite data

To ensure that amplification of alleles is consistent throughout the duration of a study, a positive control was run with every PCR batch [51]. To calculate the error rate, amplification was repeated with every chosen locus for a random subset of 10% of all samples [51], [52] using the Random Number Generator Software by Graziano & Raulin [53]. Afterwards allele sizes were compared and the percentage of mistypes was calculated.

To avoid allele scoring errors, only loci that could be scored unambiguously were included. Additionally, all loci across all populations were tested on large-allele dropout using the software MICRO-CHECKER [54].

The software INEst (Inbreeding/Null Allele Estimation) by Chybicki & Burczyk [55] was used to estimate the frequency of null alleles at microsatellite loci within a population. A feature of this program is to take the possibility of inbreeding within a population into account. In fact, inbreeding and null alleles can both cause an excess of homozygotes. The population inbreeding model in INest was used to test for null alleles. For every allele and for a possible null allele the allele frequency p_ij_ is given. An allele frequency of zero indicates no null alleles.

### Among population genetic diversity

The default diversity variables for microsatellites were calculated for each population. The web server of the software tool Genepop [56] (http://genepop.curtin.edu.au/) was used to calculate the observed heterozygosity (H_o_), expected heterozygosity (H_e_), deviations from Hardy-Weinberg Equilibrium (HWE), inbreeding coefficient F_is_ and linkage disequilibrium (LD) across all pairs of loci for each population. The test on HWE and LD are based on a Markov Chain Monte Carlo simulation (MCMC) with dememorisation 5000, batches 500 and iterations per batch 5000 [56], [57]. Genepop uses the exact Hardy-Weinberg test of Haldane [58], Guo & Thompson [57] and Weir [59], estimates the exact p-value and calculates the inbreeding coefficient F_is_ according to Weir & Cockerham [60] and Robertson & Hill [61]. The HWE and F_is_ were calculated for each population.

To determine the information content of microsatellite loci, the mean number of alleles per locus, the median of number of alleles per locus and the polymorphism information content (PIC: characterizes a locus as highly informative if PIC>0.5, reasonably informative if 0.5>PIC>0.25 and slightly informative if PIC<0.25 [62]) were calculated for each microsatellite locus with the Excel Microsatellite Toolkit [50].

In order to examine potential differences in genetic diversity between the populations the proportion of polymorphic loci (χ^2^-test), the average number of alleles per locus (Friedman test, N = 33, because one locus in the *P. taeniatus* Nigeria was not evaluable), observed and expected heterozygosities and inbreeding coefficients (Friedman test, N = 7, because only loci that were polymorphic in all three populations were included) were compared. Post-hoc, pairwise population comparisons were conducted with Wilcoxon signed-ranks tests. For statistical analysis the software R version 2.9.1 (R Development Core Team, Vienna, Austria) was used.

### Within population genetic diversity and substructure of the Moliwe population

For the *P. taeniatus* Moliwe population the default diversity variables (see above) were calculated for the six sampling sites (presented in Table S1). Due to the low sample size (N = 6), samples from site E data were not included for further analyses. The degree of genetic divergence among sampling sites of the Moliwe population was estimated pairwise with Wrights Fixation index F_ST_ using the software Arlequin, version 3.11 [63] and with D_est_ [64] using SMOGD version 1.2.5 [65].

The software *structure* version 2.3 [66] was used to evaluate the potential substructure of the *P. taeniatus* population from the Moliwe river by estimating the number of subpopulations (K). Population numbers K = 1–5 according to the five sampling sites were tested three-times at the population level based on 1,000,000 generations (MCMC) after a burn-in period of 100,000. Assuming that gene flow is possible between neighboring populations, the admixture model was applied for the ancestry of individuals using sampling locations as prior information [67]. Runs were conducted using the correlated allele frequencies model that has better power to detect subtle population structure [66], [68]. Additionally, the default diversity variables were calculated according to possible subpopulations. In some cases the number of K estimated by *structure* does not correspond to the real number of subpopulations [69]. Therefore the number of subpopulations was additionally estimated based on the approach of Evanno [69] using the software *Structure Harvester* [70]. To align the cluster membership coefficients of the three *structure* runs and to graphically display the results, the programs CLUMPP version 1.1.2 [71] and *Distruct* version 1.1 [72] were used.

A simple Mantel test was conducted using the software “zt” [73] to test for isolation by distance. Significance was estimated based on 10,000 permutations. Matrices of F_ST_ values and geographical distances (based on a GPS map of the Moliwe river, Figure 1) were compared for the five sampling sites.

The software BOTTLENECK version 1.2.02 [74], [75] was used to detect recent bottleneck events in the *P. taeniatus* Moliwe population. A Wilcoxon signed-ranks test was performed to test for heterozygote excess of the 17 microsatellite loci. The Wilcoxon signed-ranks test was conducted with all types of models (infinite allele model (IAM), stepwise mutation model (SMM) and two-phase model (TPM)). The TPM was run with the recommended settings of 95% single step-mutations, 5% multiple-step mutations and a variance among multiple steps of approximately 12. With the software BayesAss+ version 1.3 [76] the recent migration rates were calculated using the default settings.

## Results

The total amplification error rate was 2.02%. No evidence for large-allele dropout for each locus in each population was detected using the software MICRO-CHECKER. Null alleles seem to be present in the Moliwe population at locus GM120 (p_ij_ = 0.079), in the Nigeria population at loci GM271 (p_ij_ = 0.121) and GM386 (p_ij_ = 0.073) and in *P. pulcher* Nigeria at GM120 (p_ij_ = 0.053) and GM211 (p_ij_ = 0.193).

The test for genotypic disequilibrium for each locus pair of the 17 microsatellite loci was carried out for each population and across loci of the three *Pelvicachromis* populations. In most cases no linkage disequilibrium was detected. There were ten significant values (p<0.05) indicating linkage disequilibrium in the *P. taeniatus* population from Moliwe river and five significant values in the *P. pulcher* Nigeria, but these were no longer statistically significant after Bonferroni correction.

### Among population genetic diversity

The proportion of polymorphic microsatellite loci did not differ significantly between the three studied *Pelvicachromis* populations (in *P. taeniatus* Moliwe 12 out of 34, in *P. taeniatus* Nigeria 15 out of 33, and in *P. pulcher* Nigeria 22 out of 34 microsatellites were polymorphic, χ^2^-test, χ^2^ = 2.019, df = 2, p = 0.364).

**Figure 2:**
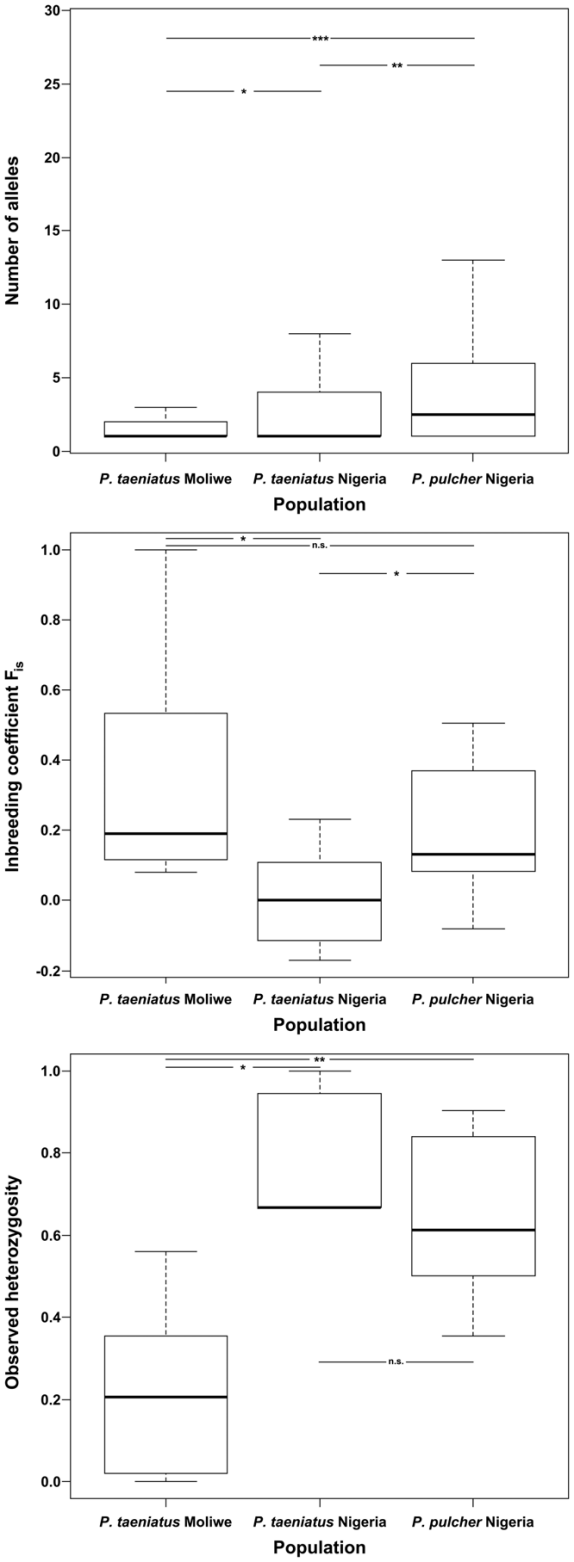
Comparisons of the three *Pelvicachromis* populations. Median (± quartiles and ranges) of (a) number of alleles per locus, (b) inbreeding coefficient F_is_ and (c) observed heterozygosity. In the analyses only loci were included that were polymorphic in all populations (N = 7). * indicates p<0.05, ** p<0.01, *** p<0.001, n.s. = not significant, p>0.05.

The populations differed significantly in average allele number per microsatellite locus (Friedman test, N = 33, df = 2, χ^2^ = 18.439, p<0.001, Figure 2 and Table 1, Table S1) with *P. taeniatus* from Moliwe having on average significantly fewer alleles per locus than the Nigerian *P. taeniatus* and *P. pulcher* populations (Wilcoxon signed-ranks test, N = 33, V = 23, p = 0.021 and N = 33, V = 27, p<0.001, respectively), even though more individuals were examined from the Moliwe river. The *P. taeniatus* and *P. pulcher* populations from Nigeria also differed significantly in allele numbers (Wilcoxon signed-ranks test, N = 33, V = 21.5, p = 0.005).

**Table 1:** Microsatellite diversity indices in the three studied *Pelvicachromis* populations.

Population	N	Loci	A	A_median_	H_e_	H_o_	P_HWE_	F_is_
*P. taeniatus* Moliwe	200	17	3.00	1.5	0.1893	0.1673	<0.001	0.116
*P. taeniatus* Nigeria	9	16	5.63	2.5	0.607	0.5486	0.3513	0.096
*P. pulcher* Nigeria	31	17	8.88	3.0	0.5736	0.4573	<0.001	0.203

Sample size (N), number of loci typed in the sample (Loci), mean number of alleles per locus (A), median of number of alleles per locus (A_median_), mean expected (H_e_) and observed heterozygosity (H_o_), results of probability test for deviation from expected Hardy-Weinberg proportions (P_HWE_), mean inbreeding coefficient (F_is_).

Expected heterozygosities (H_e_) and observed heterozygosities (H_o_) of the polymorphic microsatellite loci differed significantly between the three *Pelvicachromis* populations (Friedman tests, df = 2, N = 7, H_e_: χ^2^ = 10.571, p = 0.005, H_o_: χ^2^ = 10.286, p = 0.006, Figure 2 and Table 1, Table S1). Average individual heterozygosity was significantly lower in *P. taeniatus* from Moliwe than in *P. taeniatus* or *P. pulcher* from Nigeria (Wilcoxon signed-ranks test, N = 7, H_e_: V = 0, p = 0.016, H_o_: V = 0, p = 0.016 and N = 7, H_e_: V = 0, p = 0.016, H_o_: V = 1, p = 0.031, respectively). Between the Nigerian *P. taeniatus* and *P. pulcher* populations no significant difference was evident (Wilcoxon signed-ranks test, N = 7, H_e_: V = 10, p = 0.578, H_o_: V = 21, p = 0.297).

The inbreeding coefficients differed significantly, too (Friedman test, N = 7, df = 2, χ^2^ = 7.714, p = 0.021, Figure 2 and Table 1, Table S1). Both, *P. taeniatus* from Moliwe and *P. pulcher* from Nigeria had significantly higher F_is_ values than the *P. taeniatus* population from Nigeria (Wilcoxon signed-ranks tests, N = 7, V = 36, p = 0.047 and V = 0, p = 0.016, respectively), whereas the differences between the *P. pulcher* Nigeria and *P. taeniatus* Moliwe population were not significant (Wilcoxon signed-ranks test, N = 7, V = 16, p = 0.813). The latter populations showed a significant heterozygote deficit, whereas *P. taeniatus* from Nigeria did not (Table 1).

### Within population genetic diversity and substructure of the Moliwe population

Within the Moliwe river population, fish from the Mile 4 stream (sampling site A) showed moderate genetic differentiation in comparison to the other sampling sites (F_ST_>0.1, p≤0.001, Table 2). The genetic differentiation between sampling sites B, C, D and F was low with F_ST_ values below 0.05 (Table 2). In contrast, the D_est_ values displayed no differentiation at all (Table 2).

**Table 2:** F_ST_ values, D_est_ values and distances between the five sampling sites of the Moliwe population.

Sampling site	A	B	C	D	F
A	-	2.201	1.797	0.367	1.240
B	**0.139** *** **/**0.015	-	0.405	1.834	0.961
C	**0.109** *** **/**0.006	0.015*/0.0003	-	1.430	0.557
D	**0.137** *** **/**0.007	0.008/0.0004	0.014*/0.0007	-	0.873
F	**0.141** *** **/**0.011	-0.002/0	0.012*/0	0.010/0.0001	-

The upper matrix gives the distances between sampling points in kilometers. The lower matrix shows the estimated pairwise F_ST_ values (left) and D_est_ values (right) between the five sampling sites of the Moliwe population.

***indicates p≤0.001, and.

*p<0.05.

Results that remain statistically significant after Bonferroni correction are marked in bold.

**Figure 3:**
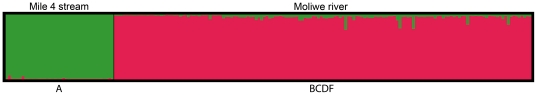
Population structure. *Structure* bar plot of the estimated membership coefficients Q for each individual in each cluster. Two subpopulations (K = 2) were estimated in the Moliwe population: Mile 4 stream (A) and Moliwe River (B, C, D, F). Each bar represents one individual (x-axis) of the Moliwe population and is broken in K colored segments, with the length proportional to each of the K inferred clusters (y-axis).

In the *structure* analysis for K = 2 the value of the estimated log-likelihood of K [LnP(D)] was maximized (LnP(D)_mean_ = −2181.2), indicating that the *P. taeniatus* Moliwe population is most likely composed of two subpopulations: sampling site A (Mile 4 subpopulation) and sampling sites BCDF (Moliwe subpopulation, Figure 3). This was also confirmed using the approach suggested by Evanno [68] with ΔK = 2 as most likely number of populations.

The single Mantel test revealed no correlation between genetic and geographic distance between Moliwe river sampling sites (r = 0.277, p = 0.15, Table 2).

Based on *structure* and F_ST_ results, we assume that sampling site A (Mile 4 subpopulation) is separated from the rest of the population and that the sampling sites B, C, D and F belong to one subpopulation (Moliwe subpopulation, see Figure 1). The Moliwe river subpopulation deviated significantly from HWE and showed a positive inbreeding coefficient. The Mile 4 subpopulation showed no significant deviation (Table 3). While in the Moliwe subpopulation 15 out of 17 loci were polymorphic, in the Mile 4 subpopulation only 3 loci were polymorphic. Detailed information on all microsatellite loci used in this study and on microsatellite diversity of the sampling sites is given in Tables S1 and S2. The Wilcoxon signed-ranks test did not detect a significant excess of heterozygosity in the Moliwe population according to the infinite allele model (IAM, p = 0.551), the stepwise mutation model (SMM, p = 0.959) and the two-phase model (TPM, p = 0.959) that was expected to best reflect the mutational process of microsatellite loci [75]. No recent bottleneck event was detected. Due to the estimated population structure, subpopulations were tested on bottlenecks as well. No bottlenecks were detected in the Mile 4 subpopulation (IAM, p = 0.438; TPM, p = 0.937; SMM, p = 0.907) and the Moliwe river subpopulation (IAM, p = 0.319; TPM, p = 0.926; SMM, p = 0.938). Migration rates calculated with BayesAss+ revealed no migration from the Mile 4 subpopulation into the Moliwe river subpopulation (m = 0.002±0.002; mean ± SD) and little migration from the Moliwe river subpopulation into the Mile 4 subpopulation (m = 0.169±0.097).

**Table 3:** Microsatellite diversity indices at the Mile 4 (sampling site A) and the Moliwe (sampling sites B, C, D and F) subpopulation.

Subpopulation	N	Loci	A	A_median_	H_e_	H_o_	P_HWE_	F_is_
Mile 4 river	40	17	1.47	1	0.0785	0.0809	0.3429	−0.031
Moliwe river	154	17	2.94	1.5	0.2120	0.1948	<0.001	0.081

Sample size (N), number of loci typed in the sample (Loci), mean number of alleles per locus (A), median of number of alleles per locus (A_median_), mean expected (H_e_) and observed heterozygosity (H_o_), results of Hardy-Weinberg probability test for deviation from expected Hardy-Weinberg proportions (P_HWE_), inbreeding coefficient F_is_.

## Discussion

The aim of the present study was to examine the degree of inbreeding in the wild population of the cichlid fish *Pelvicachromis taeniatus*, which showed kin mating preferences in the laboratory [12], [41]. Microsatellites were established towards that aim and high inbreeding in the wild population of *P. taeniatus* from Moliwe river could be suggested.

The average allelic diversity per microsatellite was lowest in the *P. taeniatus* population from Moliwe river compared to the two Nigerian *Pelvicachromis* populations. The origin of the low genetic diversity in the Moliwe population remains ambiguous. Based on our results, a recent bottleneck can most likely be ruled out. Other plausible causes might be the appearance of a bottleneck earlier in the population history [25], [77], [78], a founder effect [79] or inbreeding in combination with drift or selection. As expected by the low allelic diversity, the expected and observed heterozygosities of the polymorphic microsatellite loci were significantly lower in the Moliwe population than in the other populations. The level of heterozygosity in *P. taeniatus* Nigeria and *P. pulcher* Nigeria resembles those found in other cichlid populations [80]–[84], possibly suggesting that the low genetic diversity in the Moliwe population is a local phenomenon.

We detected significant heterozygote deficiencies (i.e. positive F_is_ values) at population level in *P. taeniatus* from Moliwe and *P. pulcher* from Nigeria, which might indicate non-random mating. Heterozygote deficiencies revealed in several studies indicate that inbreeding is quite common in fish [81], [83], [85]–[87]. The process underlying kin mating, however, cannot be directly inferred from population genetic data.

When populations are substructured even random mating within subpopulations can lead to significant deviations from HWE at population level (Wahlund effect, [88], [89]). And indeed, considering the estimation of subpopulations with *structure* and the F_ST_ values it is indicated that the *P. taeniatus* Moliwe population is substructured. Most likely, it is composed of two subpopulations: one consisting of individuals from sampling site A of the Mile 4 stream and another consisting of the remaining sampling sites along the Moliwe river itself. This population structure is also supported by the high F_ST_ values between sampling site A and the other sampling sites (F_ST_>0.1). Fish from the Mile 4 stream (A) were caught above a waterfall that fish from the Moliwe river are unlikely able to ascend. In contrast, D_est_ values indicate no population substructuring.

As significant deviations from HWE were also found in the Moliwe subpopulation (and within the Moliwe subpopulation even within sampling sites), the positive inbreeding coefficients most likely do not result from a Wahlund effect. Positive inbreeding coefficients in the wild Moliwe population are thus in accordance with the active inbreeding hypothesis derived from the laboratory experiments [12], [41]. In cichlids, population genetic studies from Pouyaud et al. [81] and Agnèse et al. [83] similarly suggest kin-biased mating preferences in the mouthbrooding tilapia *Sarotherodon melanotheron* and *Oreochromis niloticus*. The presented study clearly cannot prove that the correlation between positive F_is_ values in the wild Moliwe population and observed kin mating preferences in the lab is causal, however, it might contribute to the discussion about the origin of inbreeding in nature by stressing that inbreeding need not necessarily result from limited outbreeding opportunities. This approach is in accordance with theoretical predictions (e.g. [10], [14]) and a closer look to the literature suggests similar preferences in other animal species: three early studies in birds indicate regular close inbreeding in zebra finches, *Taeniopygia guttata*, ([90] but see [91]), in superb blue wrens, *Malurus splendens* [92], and in pukekos, *Porohyrio p. melanotus* [93]. Recently, Cohen and Dearborn [94] showed considerable genetic similarity between mates in the great frigatebird, *Fregata minor*. In contrast to the hypothesis that inbreeding should be reduced through extrapair copulations [95], Rätti et al. [96] found that in the pied flycatcher, *Ficedula hypoleuca*, breeding pairs with low genetic similarity had more extrapair young in the nest, than more related breeding pairs. Kleven et al. [97] showed that extrapair mating partners were even more closely related than the social mate in the North American barn swallow, *Hirundo rustica erythrogaster*. Besides birds, there are also examples of inbreeding preferences in amphibians [98], [99] and in the parasitic cestode *Schistocephalus solidus* [100]. Along with a considerable number of studies which found no inbreeding avoidance in different taxa [32], [101]–[107], the findings suggest that inbreeding avoidance is not as prevalent as often assumed [108]. That might be partly due to costs of outbreeding, but might also be due to inherent benefits of inbreeding.

Null alleles that can also lead to deviations from Hardy-Weinberg equilibrium can most likely also be ruled out as an explanation. Calculations with the software INest suggested possible null alleles in five microsatellite loci with allele frequencies between 0.053 and 0.193. Dakin & Avise [109] concluded that under realistic situations, when microsatellite null alleles are uncommon to rare (allele frequencies <0.2), they cause only a slight underestimate of the average exclusion probability at a locus, but probably not of sufficient magnitude to warrant great concern. At such low allele frequencies it is unlikely that null alleles influence Hardy-Weinberg equilibrium appreciably. Genotyping errors can also affect allele frequency estimates [110]. This can result in an excess of homozygotes, departure from Hardy-Weinberg equilibrium or overestimation of the level of inbreeding [111], [112]. Altogether, no significant genotyping errors were detected. With more than 200 individuals this population genetic study is comparatively large and a mean total genotyping error rate of 2.02% at 17 microsatellite loci is low. No evidence for large-allele dropout was detected either.

In conclusion, the low allelic diversity, low heterozygosity, and significant heterozygote deficiency of microsatellite loci point to a highly inbred *P. taeniatus* population in the Moliwe river. Considering previous laboratory experiments [12], [41], our model system provides unique empirical evidence for kin mating preferences. However, kin mating preferences can only be an advantageous strategy when the benefits of inbreeding override the costs, i.e. inbreeding depression, which might be true for the examined population, at least in the short term [12]. Theory predicts that recessive deleterious alleles – which when expressed in homozygotes are thought to be the main cause for inbreeding depression – can be purged from a population by selection [29], [34], [38], [113]–[118]. Glémin [38] distinguished between “purging by drift” and “purging by non-random mating”, i.e. mating between close relatives, and showed that purging by non-random mating can be particularly effective. For example, Fox et al. [119] recently showed a reduction of the genetic load of almost 75% after 3 generations of sibling matings in the beetle *Stator limbatus*. Serial mating between kin might have reduced the magnitude of inbreeding depression in the Moliwe population as well. Alternatively, passive purging by drift might have been an important prerequisite for the evolution of kin mating preferences in our study population. In any case, purging might be a plausible scenario for reducing the costs of inbreeding in the Moliwe population in particular, because purged alleles most likely cannot be reintroduced due to the large waterfall (downstream site B) which impedes immigration. Long term (negative) consequences of inbreeding are potentially severe due to reduced genetic diversity and fixation of deleterious alleles at population level. Thus, the lack of evidence for inbreeding depression [12] might be due to a generally reduced fitness of the whole Moliwe population. In order to estimate between-population variation in inbreeding depression, it would be interesting to compare the fitness of outbred crosses from inbred populations with that from larger and consequently probably more outbred populations.

## Supporting Information

Table S1 **Microsatellites used in the analysis of the three wild** ***Pelvicachromis*** **populations.** *P. taeniatus* Moliwe (N = 200), *P. taeniatus* Nigeria (N = 9), *P. pulcher* Nigeria (N = 31). Shown are the number of alleles per locus (A), expected (H_e_) and observed heterozygosity (H_o_), results of Hardy-Weinberg probability test for deviation from expected Hardy-Weinberg proportions (P_HWE_), inbreeding coefficient F_is_, polymorphism information content (PIC).
(PDF)

Table S2 **Microsatellite diversity indices at the six sampling sites of the Moliwe population.** Sample size (N), number of loci typed in the sample (Loci), mean number of alleles per locus (A), median of number of alleles per locus (A_median_), mean expected (H_e_) and observed heterozygosity (H_o_), results of Hardy-Weinberg probability test for deviation from expected Hardy-Weinberg proportions (P_HWE_), inbreeding coefficient F_is_.
(PDF)
