# Supplementary material for: Microsatellite Support for Active Inbreeding in a Cichlid Fish
Source: PLoS One. 2011 Sep 30;6(9):e24689. doi: 10.1371/journal.pone.0024689 (PMC3184091; doi:10.1371/journal.pone.0024689)
Supplement: Table S1 — Microsatellites used in the analysis of the three wild Pelvicachromis populations. P. taeniatus Moliwe (N = 200), P. taeniatus Nigeria (N = 9), P. pulcher Nigeria (N = 31). Shown are the number of alleles per locus (A), expected (He) and observed heterozygosity (Ho), results of Hardy-Weinberg probability test for deviation from expected Hardy-Weinberg proportions (PHWE), inbreeding coefficient Fis, polymorphism information content (PIC). (PDF) [file pone.0024689.s001.pdf]

Supplementary Table S1

Microsatellites used in the analysis of the three wild Pelvicachromis populations

Examining the *P. taeniatus* Moliwe population in detail (Table S1), the PIC values showed that only locus GM658 with 14 alleles was highly informative, four loci were reasonable informative, while six were slightly informative and six contained no information.

In ten loci the expected heterozygosity was higher than the observed heterozygosity. GM120, GM211, GM386, GM553, GM658, GM665 and UNH880 deviated significantly ($p < 0.05$) from HWE, while UNH855, UNH871, GM589 and UNH940 did not significantly ($p > 0.05$) deviate. The inbreeding coefficients ranged from -0.006 to the maximum value of +1. Only at locus GM589 F_{is} was slightly negative (less inbreeding than expected with random mating). The other 10 loci indicated more inbreeding than expected with random mating.

In contrast, the other *P. taeniatus* population (Nigeria) had a lot more polymorphic loci and did not significantly deviate from Hardy-Weinberg equilibrium. The *P. pulcher* population revealed the highest polymorphism information content and significantly deviated from HWE. In 12 out of 17 loci the inbreeding coefficient was positive.

The test for genotypic disequilibrium for each locus pair of the 14 microsatellite loci was carried out for each population and across loci of the three *Pelvicachromis* populations. In most cases no linkage was detected. There were ten significant values ($p < 0.05$) indicating linkage disequilibrium in the Moliwe population between GM120 and GM553, GM120 and UNH855, GM553 and UNH871, UNH855 and UNH871, GM120 and UNH880, GM553 and UNH880, GM120 and UNH940, UNH871 and UNH940, UNH880 and UNH940 and between UNH855 and GM589. Five significant values were found in the *P. pulcher* Nigeria population between loci GM658 and UNH855, GM504 and GM530, UNH855 and GM530, UNH880 and GM397, UNH855 an UNH169. After Bonferroni correction linkage between these loci was no longer significant.

Table S1: Microsatellites used in the analysis of the three wild *Pelvicachromis* populations.

	P. taeniatus Moliwe						*P. taeniatus* Nigeria						*P. pulcher* Nigeria						
Locus	A	H_e	H_o	P_{HWE}	F_{is}	PIC	A	H_e	H_o	P_{HWE}	F_{is}	PIC	A	H_e	H_o	P_{HWE}	F_{is}	PIC	GenBank acc. no.
GM120	7	0.497	0.425	0.0001	0.146	0.460	9	0.915	1	1	-0.093	0.850	17	0.934	0.839	0.0558	0.102	0.911	BV005318
GM211	2	0.010	0	0.0024	1	0.010	2	0.111	0.111	-	0	0.099	6	0.767	0.516	0.0001	0.327	0.710	BV005362
GM271	1	0	0	-	-	0	2	0.431	0.333	1	0.226	0.321	2	0.667	0	0.2001	1	0.346	BV005386
GM386	4	0.045	0.036	0.0050	0.212	0.045	6	0.778	0.667	0.3630	0.143	0.695	8	0.715	0.355	< 0.001	0.504	0.649	BV005435
GM553	3	0.312	0.285	0.0310	0.087	0.269	14	0.958	0.889	0.3991	0.073	0.894	29	0.965	0.839	< 0.001	0.131	0.945	BV005492
GM658	14	0.691	0.560	< 0.001	0.190	0.650	8	0.868	0.667	0.1390	0.232	0.786	13	0.825	0.484	< 0.001	0.413	0.784	BV005544
GM665	3	0.035	0.005	< 0.001	0.856	0.034	4	0.570	0.667	0.6128	-0.171	0.480	4	0.568	0.613	0.8415	-0.08	0.462	BV005548
UNH855	2	0.394	0.367	0.3686	0.069	0.316	1	0	0	-	-	0	9	0.707	0.516	0.0128	0.270	0.669	G68191
UNH871	3	0.284	0.280	0.7497	0.012	0.249	3	0.625	0.667	0.5589	-0.067	0.505	1	0	0		-	0	G68201
UNH880	3	0.264	0.215	0.0041	0.184	0.229	-	-	-	-	-	-	5	0.240	0.194	0.3113	0.195	0.227	G68207
UNH940	2	0.223	0.205	0.3321	0.081	0.198	8	0.882	1	1	-0.134	0.820	26	0.967	0.903	0.0117	0.066	0.948	G68242
GM589	2	0.464	0.467	1	-0.006	0.356	1	0	0	-	-	0	1	0	0		-	0	BV005586
UNH169	1	0	0	-	-	0	4	0.785	0.667	0.1619	0.15	0.686	5	0.732	0.677	0.0058	0.075	0.667	G12321
GM397	1	0	0	-	-	0	9	0.910	0.778	0.4777	0.145	0.835	4	0.449	0.516	0.6861	-0.15	0.389	BV005438
GM173	1	0	0	-	-	0	13	0.958	0.778	0.0683	0.188	0.887	13	0.855	0.807	0.3424	0.057	0.824	BV005580
GM504	1	0	0	-	-	0	4	0.778	0.333	0.0212	0.571	0.657	5	0.364	0.355	0.6422	0.025	0.336	BV005462
GM530	1	0	0	-	-	0	2	0.208	0.222	1	-0.067	0.178	3	0.153	0.161	1	-0.056	0.143	BV005478

P. taeniatus Moliwe (*N* = 200), *P. taeniatus* Nigeria (*N* = 9), *P. pulcher* Nigeria (*N* = 31). Shown are the number of alleles per locus (A), expected (H_e) and observed heterozygosity (H_o), results of Hardy-Weinberg probability test for deviation from expected Hardy-Weinberg proportions (P_{HWE}), inbreeding coefficient F_{is}, polymorphism information content (PIC).
