# Supplementary material for: Microsatellite Support for Active Inbreeding in a Cichlid Fish
Source: PLoS One. 2011 Sep 30;6(9):e24689. doi: 10.1371/journal.pone.0024689 (PMC3184091; doi:10.1371/journal.pone.0024689)
Supplement: Table S2 — Microsatellite diversity indices at the six sampling sites of the Moliwe population. Sample size (N), number of loci typed in the sample (Loci), mean number of alleles per locus (A), median of number of alleles per locus (Amedian), mean expected (He) and observed heterozygosity (Ho), results of Hardy-Weinberg probability test for deviation from expected Hardy-Weinberg proportions (PHWE), inbreeding coefficient Fis. (PDF) [file pone.0024689.s002.pdf]

Supplementary Table S2

Microsatellite diversity at the six sampling sites of the Moliwe population

We analyzed the different sample sites of the Moliwe river system separately due to potential substructuring. Significant deviations from HWE, i.e. heterozygote deficits, were found within four of the six sampling sites. Non-significant deviations were only found in the two sampling sites with extremely high homozygosity.

Table S2: Microsatellite diversity indices at the six sampling sites of the Moliwe population.

Sampling site	N	Loci	A	A_{median}	H_e	H_o	P_{HWE}	F_{is}
A	40	17	1.47	1.0	0.0785	0.0809	0.3429	-0.031
B	40	17	2.06	1.5	0.2253	0.2020	< 0.001	0.103
C	40	17	2.24	1.5	0.1902	0.1736	0.0050	0.087
D	40	17	2.06	1.5	0.2031	0.1919	0.0027	0.055
E	6	17	1.18	1.0	0.0597	0.0392	0.8559	0.343
F	34	17	2.47	1.5	0.2268	0.2154	0.0220	0.050

Sample size (N), number of loci typed in the sample (Loci), mean number of alleles per locus (A), median of number of alleles per locus (A_{median}), mean expected (H_e) and observed heterozygosity (H_o), results of Hardy-Weinberg probability test for deviation from expected Hardy-Weinberg proportions (P_{HWE}), inbreeding coefficient F_{is}.
